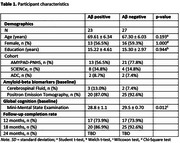# Longitudinal at‐home assessment of connected speech acoustics in Dutch amyloid‐positive cognitively normal adults

**DOI:** 10.1002/alz70856_097190

**Published:** 2025-12-24

**Authors:** Casper de Boer, Rosanne L. van den Berg, Marissa D. Zwan, Jessica Robin, John E Harrison, Roos J Jutten, Mathias Holsey Gramkow, Kristian Steen Frederiksen, Frederik Barkhof, Lyduine E. Collij, Argonde C. van Harten, Charlotte E. Teunissen, Elsmarieke van de Giessen, Wiesje M. van der Flier, Sietske A.M Sikkes

**Affiliations:** ^1^ Amsterdam Neuroscience, Neurodegeneration, Amsterdam, Netherlands; ^2^ Alzheimer Center Amsterdam, Neurology, Vrije Universiteit Amsterdam, Amsterdam UMC location VUmc, Amsterdam, Netherlands; ^3^ Faculty of Behavioural and Movement Sciences, Clinical Developmental Psychology & Clinical Neuropsychology, Vrije Universiteit Amsterdam, Amsterdam, Netherlands; ^4^ Winterlight Labs (Cambridge Cognition), Toronto, ON, Canada; ^5^ King's College ‐ Institute of Psychiatry, Psychology & Neuroscience, London, London, United Kingdom; ^6^ Alzheimer Center Amsterdam, Neurology, Vrije Universiteit Amsterdam, Amsterdam UMC location VUmc, Amsterdam, Amsterdam, Netherlands; ^7^ Metis Cognition Ltd., Kilmington, Wiltshire, United Kingdom; ^8^ Amsterdam Neuroscience, Amsterdam, Netherlands; ^9^ Danish Dementia Research Centre, Dept. of Neurology, Copenhagen University Hospital ‐ Rigshospitalet, Copenhagen, Denmark; ^10^ Department of Clinical Medicine, Faculty of Health and Medical Sciences, University of Copenhagen, Copenhagen, Denmark; ^11^ University College London, London, United Kingdom; ^12^ Amsterdam Neuroscience, Brain Imaging, Amsterdam, Netherlands; ^13^ Department of Radiology and Nuclear Medicine, Vrije Universiteit Amsterdam, Amsterdam University Medical Center, location VUmc, Amsterdam, Netherlands; ^14^ Lund University, Lund, Sweden; ^15^ Amsterdam University Medical Center (Amsterdam UMC), Amsterdam, North Holland, Netherlands; ^16^ Amsterdam Neuroscience, Neurodegeneration, Amsterdam, Noord‐Holland, Netherlands; ^17^ Neurochemistry Laboratory, Department of Clinical Chemistry, Amsterdam Neuroscience, Vrije Universiteit Amsterdam, Amsterdam UMC, Amsterdam, Netherlands; ^18^ Department of Radiology & Nuclear Medicine, Amsterdam UMC, Amsterdam, Netherlands; ^19^ Alzheimer Center Amsterdam, Amsterdam, Netherlands; ^20^ Department of Epidemiology and Data Science, Amsterdam UMC, Amsterdam, Netherlands

## Abstract

**Background:**

Automated analysis of connected speech is emerging as a promising digital biomarker of Alzheimer's disease (AD). Considering the reliance of connected speech on multiple interacting cognitive functions, fine‐grained analysis may have the potential to capture subtle cognitive deficits in the very early stages of AD. In this study, we identified reliable acoustic and linguistic speech features, and examined the association between amyloid‐beta (Aβ) pathology and longitudinal connected speech in cognitively normal elderly.

**Methods:**

We included 50 cognitively normal (age 68.4±6.8 years, *n* = 29 female, *n* = 23 Aβ‐positive) adults from three clinical cohorts at Alzheimer Center Amsterdam (Table 1). Aβ‐status was based on local cut‐offs for ptau/Aβ42 ratio in cerebrospinal‐fluid or visual inspection of amyloid positron emission tomography imaging. The testing paradigm consisted of a five‐day remote burst assessment, comprising seventeen tablet‐based speech tasks (picture description, journal‐prompt storytelling, verbal‐fluency, 10 min/day administration time), which was repeated at 2 weeks and 12, 18, and 24 months. Various acoustic‐and linguistic features (e.g. pauses, noun/pronoun use), were extracted from the voice recordings. Mean scores were calculated over the five‐day burst. The association between Aβ‐pathology and speech features was investigated using linear mixed models separately for each task.

**Results:**

Based on the repeated assessment at 2 weeks, 10 acoustic and 8 linguistic speech features were identified as having good test‐retest reliability (ICC > 0.75). At baseline, differences between Aβ‐positive and Aβ‐negative individuals in these features were generally non‐significant. However, we found trends towards a higher pause‐to‐word ratio (picture description: beta=0.05, *p* = 0.040; journaling: beta=0.07, *p* = 0.032) and higher pronoun‐to‐noun ratio (picture description: beta=0.03, *p* = 0.033) in Aβ‐positive individuals. Furthermore, we observed differences in longitudinal trajectories on various pause measures between groups, indicating stable speech metrics in Aβ‐negative individuals versus more pauses in Aβ‐positive individuals.

**Conclusion:**

Our results provide indications that Aβ‐pathology is associated with altered metrics of connected speech in cognitively healthy adults, both at a single time point and longitudinally, albeit with small effects. This supports the notion that remote multi‐day connected speech assessments have the potential to serve as a digital biomarker for subtle cognitive changes in preclinical AD, for example in observational studies and decentralized trials.